# Redefining Agricultural Residues as Bioenergy Feedstocks

**DOI:** 10.3390/ma9080635

**Published:** 2016-07-28

**Authors:** Marlon Caicedo, Jaime Barros, Bernardo Ordás

**Affiliations:** 1 Instituto Nacional de Investigaciones Agropecuarias (INIAP); Quito 170315, Ecuador; marlon.caicedo@iniap.gob.ec; 2 Department of Biological Sciences, College of Arts and Sciences, University of North Texas, Denton, TX 76203, USA; Jaime.Barros-Rios@unt.edu; 3 Misión Biológica de Galicia (CSIC), Apartado 28, Pontevedra 36080, Spain

**Keywords:** senescence, photosynthesis, chlorophyll content, climate change

## Abstract

The use of plant biomass is a sustainable alternative to the reduction of CO_2_ emissions. Agricultural residues are interesting bioenergy feedstocks because they do not compete with food and add extra value to the crop, which might help to manage these residues in many regions. Breeding crops for dual production of food and bioenergy has been reported previously, but the ideal plant features are different when lignocellulosic residues are burnt for heat or electricity, or fermented for biofuel production. Stover moisture is one of the most important traits in the management of agricultural waste for bioenergy production which can be modified by genetic improvement. A delayed leaf senescence or the stay-green characteristic contributes to higher grain and biomass yield in standard, low nutrient, and drought-prone environments. In addition, the stay-green trait could be favorable for the development of dual purpose varieties because this trait could be associated with a reduction in biomass losses and lodging. On the other hand, the stay-green trait could be detrimental for the management of agricultural waste if it is associated with higher stover moisture at harvest, although this hypothesis has been insufficiently tested. In this paper, a review of traits relevant to the development of dual purpose varieties is presented with particular emphasis on stover moisture and stay-green, because less attention has been paid to these important traits in the literature. The possibility of developing new varieties for combined production is discussed from a breeding perspective.

## 1. Introduction

The increase in the global demand for food and energy are two main challenges that the world is facing today. Most energy nowadays comes from fossil fuels which are not renewable and have a negative impact on the environment due to the high CO_2_ emissions that cause the greenhouse effect. Using plant biomass as an energy source is a sustainable alternative, as it is a renewable, has large availability, and contributes to the reduction of CO_2_ emissions [1]. Plant breeding has been successfully used to improve crops, not only to increase crop yields but also to enhance resistance and/or tolerance to biotic and abiotic stresses or specialty traits such as protein quality [2], oil content [3], or delayed senescence or stay-green (SG) [4], among other traits.

In several crops there are genotypes with delayed senescence or stay-green (SG) [5,6]. This trait has been reported to have a positive influence on the crop production and is also associated with lodging resistance, tolerance to low nitrogen environments, and drought tolerance [7,8,9]. With those qualities, the SG trait could contribute to the development of plant ideotypes for dual purpose varieties, which would produce more grain and stover per area. SG genotypes could be an excellent option for simultaneous production of food or feed from grain and bioenergy from stover [10]. Not only stover from SG genotypes, but in general, biomass from agricultural residues can be turned into useful bioenergy [11]. On the other hand, in the exploitation of vegetal biomass for bioenergy several factors that influence the cost of bioenergy production should be considered, for example, stover moisture at harvest [12,13].

From a plant breeding perspective, an introduction on how agriculture could mitigate climate change, considering different main world food crops, is presented in this review. Particular emphasis is placed on two plant traits that are less reported in the literature: (i) delayed senescence, which could help increase the overall plant biomass; and (ii) biomass moisture at harvest, with potential to reduce the transportation, the storage, and the processing costs of agricultural residues. Our aim is to encourage the establishment of breeding programs for dual purpose food-fuel in cereal crops.

## 2. Can Agriculture Help Mitigate Climate Change?

In this section, we briefly review the influence of climate change on agricultural production based on projection statistics on greenhouse gas (GHG) emissions from main world crops, predicted by international organizations such as the Intergovernmental Panel on Climate Change (IPCC), the International Energy Agency (IEA), and the Food and Agriculture Organization of the United Nations (FAO). The main groups of feedstocks currently used for bioenergy production are also addressed.

### 2.1. Climate Change and Agriculture

IPCC is the international body for assessing the science related to climate change, and it was set up in 1988 by the World Meteorological Organization (WMO) and the United Nations Environment Programme (UNEP) to provide policymakers with regular assessments of the scientific basis of climate change, as well as its impacts, future risks, and options for adaptation and mitigation. Climate change is a serious problem in the world and directly affects the population, the environment, and the global economy [14]. Climate change involves a change in the state of the climate that can be statistically monitored for a prolonged period of time (decades or longer); this change may consist of natural or artificial processes that induce alterations in land, water, and air systems. In agriculture, the negative impacts of climate change on crop yields has been reported in greater proportion compared to the positive impacts, including all aspects of food security such as production, access, and price [15,16]. Although individual locations may get a benefit from it, climate change is projected to negatively impact wheat, rice, and maize grain production in tropical and temperate regions [17]. The rise of the global temperature combined with the increase in food demand are two of the most important risks to food security nowadays, both globally and regionally [15]. The production of renewable heat, electricity, and transport fuel from plant biomass is an important component in many climate change mitigation and energy supply scenarios.

### 2.2. Emissions of Greenhouse Gases from Agriculture

In 2010, 35% of GHG net emissions were released by the energy sector, 24% from agriculture, forestry and other land use (AFOLU), 21% by industry, 14% by transport, and 6.4% by the building sector [15]. On a global level, CO_2_ emissions grew by 56% between 1990 and 2013 [18]. Over the period 1990–2010, total AFOLU net emissions increased 8% as a result of increases in agriculture emissions [19].

**Figure 1:**
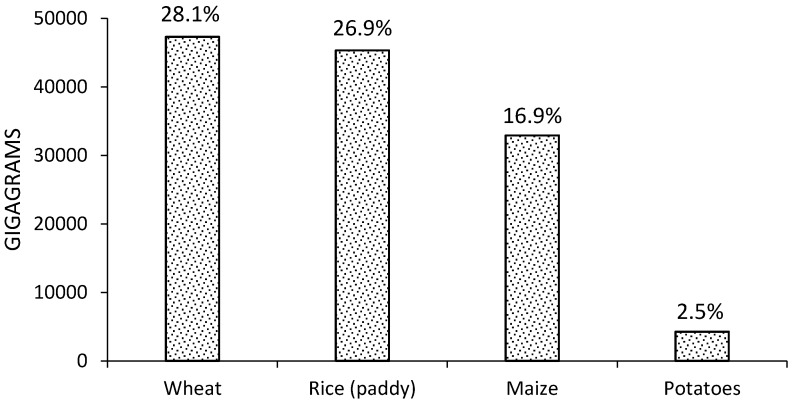
World CO_2_eq emissions by crop type from agricultural waste in four conventional crops since 1990–2012.

In 2012 FAO reported several sources of GHG emissions produced by agriculture, the most important being use of synthetic fertilizers (12%), rice cultivation (10.1%), crop residues (3.5%), cultivation of organic soils (2.8%), and burning of crop residues (0.5%) [20]. GHG emissions from crop residues consist of direct and indirect nitrous oxide (N_2_O) emissions from nitrogen in crop residues and forage/pasture renewal left on agricultural fields by farmers. Specifically, N_2_O is produced by the microbial processes of nitrification and de-nitrification taking place on the deposition site (direct emissions), and after volatilization/re-deposition and leaching processes (indirect emissions) [19]. Trace gases such as N_2_O are important for the greenhouse effect because, although they are present at concentrations lower than CO_2_, they absorb infrared radiation much more strongly than CO_2_ [21]. In the case of GHG emissions produced worldwide by agricultural waste in 2012 (Table 1), CO_2_ equivalent (CO_2_eq) emissions were ~300 times greater than N_2_O emissions for the five crops analyzed. Of these, wheat has the highest CO_2_eq and N_2_O emissions, followed by rice and maize [20]. World average CO_2_eq emission by crop type from agricultural waste since 1990–2012 (Figure 1), reveals that wheat waste produces the most CO_2_eq emission (28.1% of total crops), followed by rice and maize (26.9% and 19.6% of total crops). Wheat is also the crop with the largest area harvested (217 million hectares); however, rice produced more emissions than maize, in spite of its lower area harvested (162 million hectares vs. 179 million hectares) [20].

**Figure 2:**
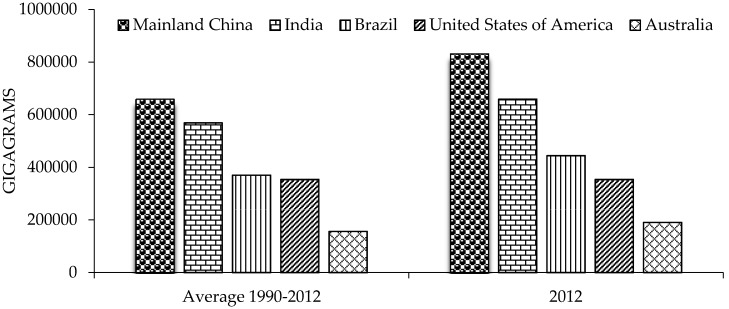
Main CO_2_eq emitters from agricultural waste reported by the Food and Agriculture Organization of the United Nations (FAO). Average 1990–2012 and 2012.

**Figure 3:**
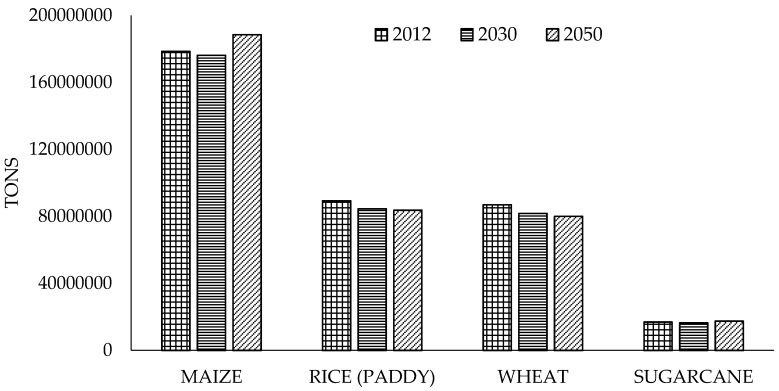
World burning of crop residues (dry matter) for 2012 and projections for 2030 and 2050.

The five largest CO_2_eq emitters from agricultural waste are China, India, Brazil, USA, and Australia. China was the greater CO_2_eq emitter during the period 1990–2012. The relative contributions of these five countries did not change over time (Figure 2) [20]. Emissions from biomass burning consist of CH_4_ and N_2_O from the combustion of biomass, and of CH_4_, N_2_O, and CO_2_ from the combustion of organic soils. With respect to world burning of crop residues, the tons burning of dry matter will be reduced in all crops analyzed by 2030, while by 2050 they will be reduced by 6.2% in rice and 7.9% in wheat; however, in maize and sugarcane it will be increased by 5.5% and 3.0%, respectively (Figure 3) [20].

These data suggest that there are important amounts of plant residues produced from modern agricultural crops that are being currently burnt to clear fields and to dispose of waste that could be potentially used as bioenergy feedstocks and thereby contribute to a global reduction of CO_2_ emissions to the atmosphere. Also, erosion and physical, chemical, and biological soil degradation would be avoided [22].

### 2.3. Plant Biomass as Energy Resource

Biomass is the result of the transformation of solar energy into chemical energy through photosynthesis. Some of this energy is stored in the plant as cellulose, hemicellulose, and lignin. Cellulose is a biopolymer composed exclusively of β-glucose molecules. Hemicellulose is a compound formed by a heterogeneous group of polysaccharides, in turn formed by a single type of monosaccharides linked by β (1–4) bonds, forming a branched linear chain. Lignin is a complex organic polymer, important in the supporting tissues of vascular plants and some algae [23]. The biomass can be turned into useful bioenergy, using crop residues, forest and wood process residues, animal wastes including human sewage, municipal solid waste (excluding plastics and most synthetics products), food processing wastes, purpose grown energy crops, and short rotation forests.

The major groups of feedstocks cover a wide range of agronomic conditions and energy production pathways including woody and herbaceous lignocellulosic plants (eucalyptus, poplar, willow, miscanthus, switchgrass) that can be grown specifically for the polymers contained in the cell walls of these plants; others groups are oil crops (soybeans, sunflower, oilseed rape, jatropha, palm oil) cultivated for seed oil, starch crops (maize, wheat) grown for seed starch, and sugar crops (sugarcane, beet, sorghum) cultivated for the sugars in stalks or underground parts [24,25]. The agricultural residuals of many crops cultivated for exploitation of seeds for feed or food are composed mainly from lignocellulosic tissue.

The use of food crops like wheat and maize for bioenergy has brought about great concern of the impact on global food security [26]. Bioenergy crops such as switchgrass or miscanthus have comparatively low resource requirements and can be grown on marginal or contaminated land (high aluminum content, salinity, and acidic soils) that is not suitable for food crop production [12]. In this case, the growth of these species does not affect world food security, but it could be difficult to guarantee that the energy crops do not displace the food crops from arable land if they are more profitable. For the major world crops, developing dual purpose commercial hybrids can be a suitable option to produce grain for human consumption, and whole plant residues for feeding livestock and/or bioenergy production. This approach would help to reduce the GHG emissions and would give an extra value to the crop, improving the income of the producers.

Conversion of lignocellulosic biomass to energy is undertaken using two main technologies: biological and thermochemical [27]. The biological conversion technologies can be divided into two main groups: anaerobic digestion by bacteria to produce methane [28] and enzymatic hydrolysis and fermentation by microbes (e.g., *Saccharomyces cerevisiae*) to produce ethanol [29]. The main thermochemical technologies can be grouped into combustion, gasification, and pyrolysis. The main characteristics of each group are summarized in Pandey et al. [30]. Combustion and gasification are performed in the presence of oxygen and partial oxygen, respectively, while pyrolysis is performed in the absence of oxygen. Biomass pyrolysis can be divided into three main types, including slow pyrolysis, which has been conventionally applied for the production of charcoal and fast and flash pyrolysis for production of bio-oils [31]. In direct combustion biomass is burned in biomass boilers to generate heat which, in turn, can be used to generate steam and power [32]. In gasification, the gaseous product obtained is usually known as syngas. The production of multiple energy products from syngas has been proposed by Heidenreich and Foscolo [33] for increasing the economic viability and sustainability of gasification.

Cogeneration is the simultaneous generation of two different forms of useful energy from a single primary source of energy [34]. Ahrenfeldt et al. [35] indicated that flexibility and efficiency are key characteristics and analyzed gasification for cogeneration processes, including production of heat-power-fuel and heat-power-fertilizers. According to Giacchetta et al. [36], combustion and gasification are only profitable for medium and large plant sizes which, otherwise, show several implementation difficulties to supply, transport, and store the large amounts of biomass that are needed to feed the plants. These authors also indicated that the cogeneration systems allow the use of small and micro scale plants which are more adequate for the exploitation of biomass, especially the solid ones, because they do not have the implementation difficulties of large plants. The use of small combined cycles for simultaneous generation of heat and power from the external combustion of solid biomass and low quality biofuels is feasible thanks to advances in designing micro steam expanders and gas-to-gas heat exchangers [37]. Amirante et al. [38] demonstrate the feasibility and convenience of a trigeneration system feeding with olive tree pruning residues, which produces cooling, heating and electrical power and is capable of satisfying the entire thermal and cooling demands of an airport in Italy as well as part of the electrical energy required by the airport. Borsukiewicz-Gozdur et al. [39] presented proposals of the organic Rankine cycle (ORC) power plant solutions to be applied for utilization of wood waste to produce electricity and heat. Prando et al. [40] assessed the energy performance of a cogenerative system consisting in a biomass boiler coupled with an ORC generator under real operating conditions and identified potential improvements.

Although the energy conversion technologies have been classified in biological and thermochemical terms, it is possible to integrate both types to increase the efficiency. For example, gasification can be used to convert a solid residual—generated in the production of bioethanol by a biological conversion technology—into gaseous fuels from which electricity, liquid fuels, and chemicals can be produced [41]. It has been also proposed to integrate the different types of thermochemical technologies; for example, Yuan and Eden [42] studied a novel biorefinery plant which integrates fast pyrolysis and gasification to produce premium quality liquid fuels and propylene.

Patel et al. [43] indicated that selection of technologies is highly dependent on the feedstock used, end products, and geographical location, and concluded that more research is required to optimize the process conditions in order to maximize the bioenergy production. In addition, the characteristics of the biomass could also be improved to optimize the bioenergy production, either by selection of appropriate species and/or by genetic improvement within species. In the case of exploitation of agricultural residuals, the replacement of species to produce a more appropriate feedstock for bioenergy conversion is not an option as this could affect the food supply. Alternatively, it is possible to implement genetic improvement of the crops that are being cultivated for improving their characteristics for the double exploitation (food and bioenergy).

## 3. Dual Purpose Crops Optimized for Grain-Biomass Production

This section describes the genetic advance which crops have undergone over time to increase yield and nutritional quality of grain by conventional breeding; subsequently, the added value developed in these crops by improving yield and quality of the stover, without affecting grain production, is analyzed. Finally, aspects related to the conversion of stover, transport processes of nutrients, and plant defense are analyzed, as well as the factors that influence the simultaneous production of stover and grain.

### 3.1. Preserved Crop Nutritional Quality

Crops have been improved since they were domesticated, but in the twentieth century there was an acceleration in their improvement due to the application of the scientific method and the development of successful innovations as the development of hybrids [44]. Subsequently, the use of biotechnology and molecular tools have allowed a more efficient and rapid development of superior varieties [45]. Currently, several authors have highlighted increases in stover production [46]; however, improvement in this trait should not interfere with yield and grain quality to guarantee food security.

Grain quality is related to its end use (seed, malting, baking, oil industry, etc.) and can be determined by external features (color, size, and hardness) as well as by its internal properties, such as chemical composition and nutritional value [47]. For example, maize has a higher nutritional value compared to rice and wheat, and is richer in fat, iron, and fiber content; however, it is low in protein content. About 50% of the proteins are composed of zein, which has a low content of essential amino acids, particularly lysine and tryptophan [47].

As indicated in Figure 3, the three major crops in the world are cereals, which generate a significant amount of waste that is burned. Therefore, in the breeding programs of these crops it would be particularly valuable to include stover properties as secondary improvement traits, without affecting the main goal that is feed or food production. There are also traits associated with quality grain or related to tolerance to pests, weed, and diseases which should be considered in any breeding program, either for grain or for dual-use.

### 3.2. Enhanced Biomass Conversion Qualities

The success of the bioenergy industry relies on adequate supply of high-quality biomass, ensuring that the feedstocks delivered in the biorefinery meet the physical and chemical quality specifications. The cell walls’ polymers, lignin and carbohydrates such as cellulose and hemicellulose, are the main chemical variables that control the suitability of plant biomass for conversion into liquid fuels. Lignin, cellulose, and hemicellulose are physically and chemically bound together in plant cell walls. Lignin makes up approximately 15%–25% of the dry weight of grass biomass and is the main limiting factor in the biochemical conversion of plant biomass to fermentable sugars [27]. Concomitantly, lignification is essential for plant development and one of the main mechanisms of plant defense [48]. Lignin—considered a by-product and burnt to generate heat in most pulp factories—is starting to be used directly as a raw material for industrial applications such as chemicals, plastics, or carbon fibers, among others [49]. Cellulose is the main structural component of plant cell walls and the most abundant organic material on earth, making up approximately 30%–50% of grass biomass dry weight [50]. Cellulose is currently processed to produce bioethanol, but also materials such as cellophane, cardboard, paper, and cellulose ethers such as acetate, rayon, and nitrates [51]. Unlike cellulose that contains only one sugar (glucose), hemicellulose can include several sugar monomers such as xylose, hexose, mannose, and galactose. The main hemicelluloses detected in the cell walls of monocots plants are glucuronoarabinoxylans which contains a xylose backbone with side branches containing glucuronic acid and arabinose. Hemicellulose makes up 10%–40% of the dry weight of grass biomass and is mainly used as raw material for packaging films, as a thickening, stabilizing, and gelling agents in food, and is also fermented for the bioenergy industry to be converted into liquid bioethanol [52].

The ideal plant features are different when lignocellulosic residues are burnt for heat or electricity, or fermented for biofuels production. When biomass is burned, increased lignin content is a desirable characteristic, since lignin yields more energy when burned than cellulose [53]; however, lignocellulose bioconversion by microbial fermentation requires an enhanced sugar release provided by a higher carbohydrate content and a reduced deposition of lignin. Lignocellulose bioconversion by microbial fermentation is typically preceded by a thermochemical pretreatment step to facilitate the enzymatic hydrolysis of cellulose. Substances formed during the pretreatment of the lignocellulosic feedstock, such as furan aldehydes and aliphatic acids from carbohydrate degradation or phenolic compounds from lignin degradation, inhibit enzymatic hydrolysis as well as microbial fermentation steps. Therefore, from the chemical composition perspective, the optimal bioenergy feedstock must also consider a reduced generation of fermentation inhibitors during pretreatment. The types (hexoses vs. pentoses) and levels of sugars present in cell wall carbohydrates also influence the quality of the biofuel feedstock and are connected to the efficiency of the microbial fermentation. There is extensive research aiming to modulate the deposition of carbohydrates and lignin in plant cell walls [54], as well as providing new microbial strains for enhanced fermentation and/or reduced inhibition [55] and improving or developing new bioconversion processes [56]. In the current review we focus our analysis on several agronomic traits that could improve the quality of the biomass regardless the bioconversion properties, being simultaneously compatible with the production of food, and useful as selection traits in a breeding program for a dual-use crop.

### 3.3. High Biomass Yield

Crop phenology is the most important single factor determining adaptation and crop performance [57]. For example, the flowering time has been critical in the adaptation of maize to growing seasons of different duration [58]. The life cycle of annual crops, such as maize, can be divided into a vegetative phase (from sowing to flowering) and a phase of grain filling (from flowering to physiological maturity of the grain). After grain filling, a period of field grain dry down is usually needed to diminish the moisture of the grain at harvest. There is genetic variation for the duration of the vegetative phase, the grain filling phase, and the length of the period of dry down (for example, in maize see references [59,60,61]). Genetic modification of the total duration of the crop cycle allows adaptation of the varieties to growing seasons of different duration. For a specific duration of the crop cycle, extending the duration of the vegetative phase increases grain and stover yield, but reduces the duration of grain filling and/or dry down. This results in an increment of grain and stover moisture at harvest. Thus, there is a tradeoff between grain or stover yield and moisture at harvest. Simultaneous to grain filling, foliar senescence takes place with an important role in the recycling of nutrients to the grain [62]. Delayed senescence or stay-green (SG) is the general term given to a genotype in which senescence is delayed compared to a control general pattern [6]. SG can be cosmetic or functional. In cosmetic SG phenotypes, only the green color is maintained as plants have lost their photosynthetic capacity, while functional SG refers to a phenotype which keeps capacity for photosynthetic activity. Thomas and Smart [5] divided SG into four types: type A refers to a delay in the start of senescence that proceeds at constant rates; type B refers to slow rates of senescence; type C is characterized by retention of chlorophyll, but normal loss of CO_2_ fixation capacity; and type D is characterized by an unlimited color retention that is produced by killing the leaf by freezing, boiling, or drying. Later on, Thomas and Howarth [6] added the type E that is applied to genotypes that are SG at maturity just because they are greener at flowering.

Some of the most important features that define the properties of the biomass are the characteristics of the cell wall which are affected by maturation, which occurs not only at the level of whole plant, but also at the level of individual cells. When the plant matures, the cell wall fortifies and increases its strength. Genotypes with delayed senescence or SG could have cell walls less fortified than genotypes with standard senescence. However, empirical data to corroborate this hypothesis are needed. The term SG is also used for the trait itself and is desirable for crop improvement as it is positively associated with lodging resistance and biomass yield [5]. SG is also considered a valuable trait for plants cultivated in drought/nitrogen-limited environmental conditions [63]. This trait has also been associated with resistance to several diseases, such as *Helminthosporium turcicum*, maize dwarf mosaic potyvirus, maize black streaked dwarf virus, and stalk rot (*Stenocarpella maydis*) in maize [64], spot blotch (*Bipolaris sorokiniana*) in wheat [65], charcoal rot of grain (*Macrophomina phaseolina*), *Fusarium* spp., and *Colletotrichum* spp. in sorghum [66], and *Rhyncosporium*, *Sarocladium*, and *Helminthosporium* in rice [67]. After evaluating closely related sorghum hybrids varying in rate of leaf senescence under water limiting regimes, Borrell et al. [4] demonstrated that SG hybrids produced 47% more post-anthesis biomass than their senescent counterparts under a water deficit regime, indicating that SG is a valuable trait for breeding crops, particularly grown under water limited conditions. Because of its importance and correlation with the traits described above, SG has been studied in several crops such as maize, sorghum, oats, rice, wheat, grasses, soybeans, trees, and others (Table 2).

This latter literature addresses the direct and positive correlation between SG and other agronomic traits such as grain yield, biomass yield, and drought tolerance. Therefore, from the point of view of genetic improvement, it is theoretically feasible to carry out multitrait selection to obtain dual purpose varieties. To carry out the multitrait selection, different aspects have to be considered, such as the type of inheritance of the traits (monogenic or polygenic) and the mode of plant reproduction (self-pollinated, cross-pollinated). The major challenge for breeders are those traits having negative correlations with grain yield, something that has been sometimes reported for resistance to pest attack [112].

Since the 1990s, multiple authors reported quantitative trait loci (QTL) mapping for SG and chlorophyll content traits in sorghum [113], for green leaf area and SG in maize [71], for cumulative chlorophyll content in rice [99], and for SG in wheat [114]. More recently, some other studies have considered candidate genes and discovered QTL associated with total biomass accumulation and/or grain production in bioenergy grass species including maize and sorghum [115]. On the other hand, several SG mutants (involved in functional and cosmetic SG) have been characterized in different plant species, with the most studied mutants being type C (cosmetic SG), due to the fact that chlorophylls are retained in senescent leaves (green appearance) while their photosynthetic capacity decreases [116]. These genes have been named Stay Green Rice “SGR” [100], Non Yellowing “NYE” [117], Green-Flesh “GF”, and chlorophyll retainer “cl” [103]. NYE, GF, and cl are homologs and members of the SGR gene family which plays a key role in regulating chlorophyll degradation during senescence in plants [100,117]. Schelbert et al. [118] reported another SG gene named Arabidopsis mutant deficient in pheophytinase (PPH) while Wei et al. [119] have identified another SG gene named non-yellowing (*FaNYE1*) in tall fescue, that is a homologortholog gene of *AtNYE1*. For many cosmetic SG mutants, including green cotyledon pea variety, molecular defects were recently identified in orthologous SG genes [84]. Various studies have also reported mutations named chlorophyll retainer (cl) in the SGR gene, caused by inhibition of chlorophyll degradation in both natural and dark-induced senescence in pepper [120], tomato [103], rice [121], arabidopsis [79], and pea [122]. Also, other mutations have been reported in several other genes which encode chloroplast proteins which produce functional and cosmetic SG phenotypes [83]. In addition, some families of transcription factors such as NAC (NAM, ATAF1,2, CUC2), WRKY, MYB, C2H2 zinc-finger, bZIP, and AP2/EREBP have been related to SG [123]. Thomas and Ougham [63], reported that the grain protein content (GPC) is related to a NAC transcription factor regulating cereal leaf senescence and determining the partitioning of N and minerals between the grain and crop residue; further, variations in these genes are likely to account for a range of agronomically important SG phenotypes. Although there is much literature on genetic studies of traits related to SG, there are few reports of breeding programs that have used SG as a trait selection to produce both grain and biofuels, leaving an opportunity to start work in this regard. During the change of the vegetative phase to reproductive, translocation of water, sugar, and monosaccharides (long-distance transport and slower movement) occurs from sources (usually leaves, tubers, corms, rhizomes) to sinks (growing meristems of roots and stems, fruits, and seeds) [124]. Generally, sinks are provisioned from the nearest sources; for example, in the grains of maize a large proportion of the sugars derive from the leaves surrounding the ear. For cereals, the sink is the grain, which is formed by germ or embryo (high protein), endosperm (rich in starch), pericarp (outer layer covering the grain), and in some cases the shell (consisting of plant fibers). The endosperm is the deposit of starch in the grain, which consists of two glucose polymers: amylose and amylopectin, with the type and proportion of these polymers determining the endosperm grain hardness [125]. The whole process from the translocation of reserve substances (source) until the grain physiological maturity (sink) is cyclical in plants and suggests that the relationship between the SG trait and grain filling is due to a slow movement during transport of these substances, so the delayed senescence of leaves results in longer life of the plant, favoring grain filling. On the other hand, grain development depends on two sources of N: absorbed N from soil and remobilized N from vegetative tissue [126]. Also, several authors have demonstrated that the absorption of N in SG genotypes is higher than in senescent hybrids [127]; therefore, Subedi and Ma [75] concluded that the green status of the plants at physiological maturity is maintained only under high availability and unlimited supply of N.

The photosynthetic capacity of the leaves is part of the sources and the lengthening of the period of photosynthetic activity, as in SG genotypes, which implies an increase in the source strength. However, an increase in source strength cannot be translated into yield unless there is an increment in sink strength, for example, extending the period of grain filling [128,129]. Thus, foliar senescence should be analyzed together with grain filling. The grain filling period is a critical period and its maintenance, especially in the last stage of plant maturity, can influence the final grain yield [130]. Lewandowski et al. [131] mentioned that harvest time influences not only yield, but also the dry matter content, the ash concentration, and other biomass quality traits of candidate energy crops. Edwards et al. [132] reported benefits in both wood production and increasing the length of the grain filling period in other crops by altering the timing of regulatory mechanisms such as the circadian clock. The genetic variability of the duration of leaf senescence during grain filling has been shown to affect both carbon and nitrogen acquisition [133], as well as the balance between N supply and demand [134]; therefore, its unbalance, especially when influenced by environmental factors, allows the occurrence of accelerated or delayed senescence. On the other hand, Borrell et al. [94] obtained higher grain yield under stress environments in sorghum SG phenotypes which maintained post-anthesis N uptake under drought conditions. This may be due to a high concentration of leaf nitrogen and a slow remobilization of it from the leaves; therefore, the plant maintains its photosynthetic capacity for a longer period, as well as its capacity to extract N from the soil. As seen, the SG trait is physiologically related to grain filling, and this is in turn related with grain yield and biomass. However, for high yield, a crop requires proper handling and especially a good and timely vegetal nutrition. In the SG crops, nitrogen is a key element for grain filling and final yield, so it must almost always be supplied artificially due to its low availability in the soil. However, the application of nitrogen fertilizer can increase environmental pollution due to losses of nitrogen fertilizer (evaporation, infiltration, percolation, etc.); also, it will increase production costs due to the high prices of these products and, as a consequence, benefits will be reduced. In a breeding program, the main goal has always been to obtain highly productive crops; to do so, the breeder could use the existing genetic variability for the SG trait and exploit positive correlations with other traits to develop cultivars that have more grain yield, a high biomass production, and drought tolerance among other traits.

### 3.4. Reduced Grain/Biomass Moisture at Harvest

At the genetic level, several studies have been reported for grain dry down, for example, Sala et al. [135] detected 10 QTLs associated with grain moisture as well as 8 QTLs associated with grain drying rate. Likewise, Sweeney et al. [136] reported that grain moisture and grain drying rate can be improved by selection in maize. Stover moisture is one of the most important traits in the management of agricultural waste in biofuels production, influencing harvest, storage and transportation [137]. In contrast to the abundance of works on grain dry down and grain moisture at harvest, there are few studies about stover moisture and dry down. In maize, the grain moisture typically ranges from 20% to 30% at harvest while stalk moisture is about 65% at harvest, according to Shinners et al. [138]. Similarly, Shinners and Binversie [139] reported an average ratio of 2.1:1 for total maize stover: grain moisture. However, the plant moisture at harvest depends critically on the duration of the crop cycle of the genotype, which in turn depends on the duration of vegetative phase (determined by flowering time), grain filling, and dry down. The genetic modification of the duration of the vegetative phase and grain filling would modify the moisture of the plant at harvest; however, a reduction in moisture will generally be accompanied by a decrease in both grain and stover yield. The most appropriate grain or stover moisture at harvest depends on the particular use of the grain or stover; for example, if only the grain is exploited in maize then the grain moisture should be low (14%–15%), but if the whole plant is exploited for forage then the maximum dry matter yield is obtained when the grain moisture is 45%–50% [140]. The biomass collected with higher moisture occupies more volume and, for that reason, it is more expensive to collect, store and transport [141]. Furthermore, stover harvested with high humidity allows the presence of moldy and saprophytic fungi, facilitating their rapid decomposition and, consequently, bringing about economic losses [142]. Thus, it could be imperative to dry the biomass prior to transport to the site of its conversion, although this increases the economic cost and decreases the net energy obtained because some energy has to be spent in drying the biomass. Even the dried stover may present some problems of management as it is prone to spontaneous combustion [143]. For some uses as forage or biogas maize the biomass is usually ensiled, and for silage preparation stover with high moisture (70%) is required [144]. Therefore, for maize intended for forage or biogas it is often recommended to choose varieties that flower later than those recommended for grain maize; this is because they are more productive, and high moisture is not detrimental for those particular uses [145]. White et al. [146] evaluated temperate by tropical maize hybrids—which have delayed flowering due to sensitivity to photoperiod—and recommended these kinds of hybrids for producing cellulosic ethanol. Biomass with high moisture content is suitable for biological conversion, as fermentation or microbial digestion, but biomass with low moisture is required in many combustion systems, and many biomass gasifiers are designed to operate on very low moisture content [147].

Not only the duration of the grain dry down determines the moisture of grain at harvest, but also the rate of grain dry down [61]. Similarly, the rate of stover dry down determines the moisture of stover at harvest. In maize, for example, Leask and Daynard [148] determined that stover dried at a mean rate of 1.0 g of water loss per 100 g fresh weight per day. These authors concluded that stover moisture was markedly affected by both time and atmospheric conditions, but there was also a genetic component which made it possible to select simultaneously for increased stover yield and moisture without changing grain yield and moisture at harvest. Also, reported low or non-significant correlations between stover and grain traits means that the change in stover traits through breeding do not affect grain traits. Barros et al. [149] found wide genetic variation for the rate of stover dry down in a sample of diverse germplasm of maize. They also found a moderate correlation between stover and grain moisture and suggested that the genetic improvement of the stover dry down is possible independently of the grain dry down.

For dual purpose varieties grain moisture should remain low; varieties with late flowering or sensitive to photoperiod, as those recommended by some authors for biogas or cellulosic ethanol, are not suitable. For the development of dual purpose varieties by genetic improvement, the yield of grain and stover should be increased, while the grain moisture should be decreased if it is high. Regarding the moisture of the stover, the objective of breeding will depend on the use of the biomass, although generally it would be needed to decrease moisture, particularly for thermochemical conversion processes. The SG genotypes probably maintain a higher moisture in the stover than genotypes with standard senescence because, when the grain is drying down, their leaves remains green for a longer period. Therefore, SG genotypes could be adequate for dual purpose uses as biogas production, but not for thermochemical conversion. However, more empirical data are needed about the SG trait in relationship to different energetic uses.

Shinners and Binversie [139] reported that stover yield decreased with time primarily because of losses of leaves, husk, and the top half of the stalk due to wind or other adverse weather conditions that are typical at the end of the growing season in annual crops as maize. Wilhelm et al. [150] also found that stover losses during grain dry down resulted in an increase in the harvest index. One possible advantage of the SG genotypes for the development of dual purpose varieties that has not been tested is the reduction of biomass losses and lodging because its tissues remain turgid during the last period of the growing season.

Pordesimo et al. [143] reported distribution of dry matter percentages in different parts of the maize plant (grain, stalk, leaf, cob, and husk) when grain moisture was 30.6% (at physiological maturity) and 13.0% (after grain dry down) (Table 3) and confirmed the feasibility of using a 1:1 ratio for estimating stover dry matter from grain dry matter. The distribution of the dry matter in the different parts of the plants can influence the most appropriate energetic use for the stover. For example, the maize cobs have a high heating value and could be a good solid fuel, while maize stalks have a medium or a low heating value and could be suitable for gasification at high temperature [151]. However, there is little information about the genetic variation for the distribution of dry matter in the different parts of the plants or how the distribution of the dry matter can be modified by genetic improvement. This knowledge would be valuable to assess the possibility of increasing, by genetic improvement, the percentage of dry matter in cobs in order to obtain double purpose varieties of maize which produce grain and solid fuels.

The energetic use of the stover is also conditioned by the characteristics of the cell wall; for example, a high strength or low digestibility of the cell wall is detrimental when the conversion to energy is biologically based on fermentation or microbial digestion, but not when the conversion is thermochemical to produce syngas, synthetic fuels, etc. Cone and Engels [152] indicated that an increase in lignin content results in decreasing cell wall digestibility. However, other authors de Leon et al. [153] found that breeding for increasing dry matter digestibility have not altered cell wall lignification. Barros et al. [149] also did not find a relationship between lignin content and bioethanol conversion and concluded that tissue anatomy and/or additional cell wall chemical properties may be important factors that influence production of maize stover ethanol. Several authors found genetic variation for cell wall digestibility or stover ethanol production in maize [154,155] indicating that the characteristics of the stover can be changed to improve the biological conversion to produce bioenergy. However, there is less information on how to modify the characteristics of the cell wall to improve the thermochemical conversion. The SG characteristic, if associated with a less fortified cell wall, could then be favorable for biological conversion, but indifferent to thermochemical conversion.

On the other hand, in developing dual purpose crops, grain quality is another important aspect. Besides, it should be taken into account that grains and seeds of different crops are also considered feedstock for the production of chemicals and biofuels. For example, the starch of maize grain has been used in the agrochemical industry for cosmetics, detergent, food, medical, paper, pharmaceuticals, plastics, and textile [156] and presently is used for first generation biofuel production, consequently creating problems of competence with food production [157]. Regarding the composition of grain, the SG genotypes could have an incomplete transference of nutrients from senescent leaves. Thus, Uauy et al. [158] identified a transcription factor in wheat that accelerates senescence and increases nutrient remobilization from leaves to developing grains, which results in an increased protein in the grain. A delayed senescence can also decrease the transference of nutrients to underground rhizomes in perennial grasses which is essential for growth of the following season [63].

Maize stover produced after grain harvest depends on, among other things, the genotype cultivated, the impact of environmental factors during the growing season and thereafter, the cutting height of the combine, and the drying time in the field. Based on the above, it is difficult to recommend model plants meeting all the features required for any purpose of improvement. In conclusion, it is necessary to study in SG plants the relationship between cell wall composition before and after drying (to see how it affects moisture), as well as quality grain characteristics in order to define an appropriate breeding program for dual purpose. Also, it has to be considered that wet conversion processes such as fermentation are often more suited to biomass with a higher moisture content (e.g., maize, sugarcane, barley straw) and dry conversion processes such as pyrolysis, gasification, and combustion are more suited to biomass with a lower moisture content (e.g., wheat straw, pine, switchgrass, etc.).

## 4. Conclusions

The practice of agriculture indirectly generates biomass that is not exploited and therefore the production of such agricultural residues do not represent any additional environment or economic cost to the agricultural activity mainly aimed to produce food. The exploitation of the agricultural residues to produce energy can contribute to mitigation of the greenhouse effect because the residues were generating by plants from atmospheric CO_2_. The technologies to obtain energy from biomass have been continuously progressing to optimize economic and environmental efficiency. In addition to conversion technologies, the characteristics of the biomass itself can be altered by genetic improvement to favor the conversion to energy. In the case of agricultural residues this supposes the development of dual purpose varieties which can be simultaneously exploited for food and bioenergy. Among the traits to be improved, apart from grain and stover yield, the reduction of stover moisture usually favors storage and transport as well as the thermochemical conversion. The stay-green trait (a longer duration of photosynthesis) could be, in general, favorable for the development of dual purpose varieties if it is confirmed that it is associated with lower losses of stover, mainly leaves and upper parts of stalk, and lower lodging in the final period of the growing season. However, the maintaining of green leaves could be associated with higher stover moisture at harvest which, in turn, is detrimental for thermochemical conversion. It is very important to diversify the sources of feedstock for the biofuels industry due to the climate change and the presence of different environmental conditions in the world; also, this diversification will increase the possibilities to the industry, contributing significantly to the fuel supply, and increasing the ability of the industry to serve the political goals of energy security.

## Figures and Tables

**Table 1:** World CO_2_ equivalent (CO_2_eq) and N_2_O emissions produced by agricultural waste in five conventional crops.

CROP	CO_2_eq Emission (Gg) ^1^			N_2_O Emission (Gg)		
	Direct	Indirect	Total	Direct	Indirect	Total
Wheat	42,510	9565	52,075	137	31	168
Rice paddy	42,114	9476	51,590	136	31	166
Maize	35,455	7977	43,432	114	26	140
Potatoes	3795	854	4649	12	3	15
Dry beans	1652	372	2023	5	1	7

^1^ Gigagrams.

**Table 2:** Traits related to stay-green (SG) reported by several authors in different crops.

Species	Traits Associated with SG	Reference
Maize	High grain yield, drought tolerance and low Nitrogen	[8]
	Cell-death mechanisms	[68]
	Drought tolerance, greater leaf greenness	[9]
	High yield and increase dry matter	[69]
	High leaf chlorophyll concentration, low Nitrogen	[70]
	Longer green leaf area after flowering	[71]
	Delayed leaf Senescence, higher dry matter and high sucrose accumulation	[72]
	Days to silking emergence	[73]
	Greater leaf chlorophyll content	[74]
	Greater leaf chlorophyll content, high dry matter accumulation, low N uptake	[75]
	Drought tolerance, delayed flowering, grater leaf area index	[76]
	Lower canopy senescence, longer post-silking, high C and N accumulation and yield	[77]
Maize, Rice, Soybean	High leaf N content, high CO_2_ assimilation rate, high photosynthesis rate, and greater biomass accumulation	[78]
Arabidopsis	Reduced chlorophyll degradation	[79]
	Delayed leaf senescence	[80]
Arabidopsis, Maize, Wheat, etc.	Delayed leaf senescence, reduced chlorophyll degradation, high yield and quality	[81]
	Delayed leaf senescence, longer photosynthesis duration, increase biomass production	[82]
	Delayed leaf senescence, reduced chlorophyll breakdown.	[83]
	Reduced chlorophyll and protein degradation	[84]
Wheat	High yield, high biomass production	[46]
	High photosynthetic rate, high stomatal conductance, high photochemical quenching of PSII, greater grain filling.	[85]
	High photosynthetic rate, high chlorophyll content, high malondialdehyde content, high activity of both superoxide dismutase and catalase, greater grain filling and delayed flag leaf senescence, high seed weights and per-plant yield	[86]
	Spot blotch resistance, green coloration (chlorophyll) of flag leaf, greater leaf area under greenness	[65]
	Greater leaf area under greenness, heat tolerance, high grain and biomass yield.	[87]
	Drought and heat tolerance, high vegetation index, greater grain filling	[88]
	Drought tolerance, higher grain filling rate, longer grain filling, high grain yield, high harvest index, greater grain weight and grain number per spike.	[89]
	High yield and biomass production, increase thousand grain weight	[90]
	Higher green leaf area, high grain filling, high yield	[91]
	High photosynthetic rate, high chlorophyll content, better cellular redox state of the flag leaf	[92]
	High grain yield, greater thousand grain weight, higher root length, higher root density and root weight, and slow flag leaf drying	[93]
	Low N, high yield, higher grain filling, high biomass production	[94]
	Drought tolerance, reduction in canopy size, higher root growth, grain filling and grain yield	[95]
	Greater leaf chlorophyll content, higher grain filling and grain yield.	[96]
Rice	Greater chlorophyll and N content, high yield, *Rhyncosporium*, *Sarocladium*, and *Helminthosporium* resistance	[67]
	Greater seed-setting rate, increases grain yield, grain filling and chlorophyll content	[97]
	High chlorophyll content, less chlorophyll degradation	[98]
	Retention of the green area of the flag and second leaves, high yield.	[99]
	Less chlorophyll breakdown and degradation of pigment-protein complex.	[100]
Barley	Strong winter hardiness, resistance to shattering and barley yellow mosaic virus, latter growing period, high forage dry matter yields, high grain yield	[101]
	Starch biosynthesis and quality in grain, drought tolerance, high grain filling and yield	[102]
Tomato and Pepper	Inhibition chlorophyll and protein degradation during fruit ripening	[103]
Tomato	Inhibition chlorophyll degradation	[104]
Sunflower	Greener stems at physiological maturity, low harvest seed moisture content, drought tolerance.	[105]
	High oil content, increase biomass, higher grain number and yield, resistance to stalk breakage	[7]
Broccoli	Delayed senescence, reduced chlorophyll degradation	[106]
Kiwi	Higher Pigment biosynthesis and reduced pigment degradation	[107]
*Lolium/Festuca* grasses	Slow chlorophyll catabolism	[108]
*Miscanthus*	Drought tolerance, delayed leaf senescence, increase biomass	[109]
Cassava	Drought tolerance, increase the total fresh biomass, higher root dry matter	[110]
Cowpea	Increase seed size and grain yield, heat tolerance	[111]

**Table 3:** Dry matter distribution in whole maize plant during and after physiological maturity, data reported by Pordesimo et al. [143].

Plant Parts	Percentage of Dry Matter in Plant Maize	
	Grain Physiological Maturity	After Grain Physiological Maturity
Grain	45.9	56.8
Stover	54.1	43.2
Stalk	27.5	22.0
Leaf	11.4	9.1
Cob	8.2	6.6
Husk	7.0	5.6
Grain moisture (%)	30.6	13.0
